# Supplementary material for: FERN – a Java framework for stochastic simulation and evaluation of reaction networks
Source: BMC Bioinformatics. 2008 Aug 29;9:356. doi: 10.1186/1471-2105-9-356 (PMC2553347; doi:10.1186/1471-2105-9-356)
Supplement: Additional file 1 — FERN distribution, Version 1.3. This archive contains the FERN source code and binaries as well as documentation and example models in FernML and SBML. [file 1471-2105-9-356-S1.zip › fern/doc/javadoc/fern/network/NetworkLoader.html]

NetworkLoader


---


|  |  |  |
| --- | --- | --- |
| **Overview** \| **Package** \| **Class** \| **Use** \| **Tree** \| **Deprecated** \| **Index** \| **Help** | |  |
| **PREV CLASS**   **NEXT CLASS** | **FRAMES**    **NO FRAMES**     **All Classes** |  |
| SUMMARY: NESTED \| FIELD \| CONSTR \| METHOD | DETAIL: FIELD \| CONSTR \| METHOD |  |


---


## fern.network Class NetworkLoader

```
java.lang.Object
  fern.network.NetworkLoader
```

---

``` public class NetworkLoader extends Object ```

---

**Constructor Summary**

`NetworkLoader()`


| **Method Summary** | |
| --- | --- |
| `static void` | `addTypesToFileChooser(JFileChooser dialog)`             Adds available types of networks to a `JFileChooser`. |
| `static String[][]` | `getAvailableTypes()`             Gets types of networks that are readable by FERN. |
| `static Network` | `readNetwork(File file)`             Tries to read the given file and returns the network in it (if there is one) |

**Methods inherited from class java.lang.Object**

`clone, equals, finalize, getClass, hashCode, notify, notifyAll, toString, wait, wait, wait`

**Constructor Detail**

### NetworkLoader

```
public NetworkLoader()
```


**Method Detail**

### getAvailableTypes

```
public static String[][] getAvailableTypes()
```

:   Gets types of networks that are readable by FERN. They are returned as String[N][2]
    containing for N types a description and the file pattern.

    :   **Returns:**: readable file types

---


### readNetwork

```
public static Network readNetwork(File file)
                           throws IOException,
                                  JDOMException,
                                  FeatureNotSupportedException,
                                  ClassNotFoundException
```

:   Tries to read the given file and returns the network in it (if there is one)

    :   **Parameters:**: `file` - network file **Returns:**: network object **Throws:**: `IOException` - file could not be found: `JDOMException` - file could not be parsed correctly: `FeatureNotSupportedException` - file contains a not supported sbml feature: `ClassNotFoundException` - the class SBMLNetwork could not be found

---


### addTypesToFileChooser

```
public static void addTypesToFileChooser(JFileChooser dialog)
```

:   Adds available types of networks to a `JFileChooser`.

    :   **Parameters:**: `dialog` - the `JFileChooser`


---


|  |  |  |
| --- | --- | --- |
| **Overview** \| **Package** \| **Class** \| **Use** \| **Tree** \| **Deprecated** \| **Index** \| **Help** | |  |
| **PREV CLASS**   **NEXT CLASS** | **FRAMES**    **NO FRAMES**     **All Classes** |  |
| SUMMARY: NESTED \| FIELD \| CONSTR \| METHOD | DETAIL: FIELD \| CONSTR \| METHOD |  |


---
